# Lack of evidence for a genetic association between *FGF20 *and Parkinson's disease in Finnish and Greek patients

**DOI:** 10.1186/1471-2377-5-11

**Published:** 2005-06-20

**Authors:** Jordi Clarimon, Georgia Xiromerisiou, Johanna Eerola, Vanesa Gourbali, Olli Hellström, Euthimios Dardiotis, Terhi Peuralinna, Alexandros Papadimitriou, George M Hadjigeorgiou, Pentti J Tienari, Andrew B Singleton

**Affiliations:** 1 Molecular Genetics Unit, Laboratory of Neurogenetics. National Institute on Aging, National Institutes of Health, Building 35 Room 1A1000. Bethesda, MD 20892 USA; 2 Neurogenetics Unit, Department of Neurology, Medical School, University of Thessaly, Larissa, Greece; 3 Department of Neurology, Helsinki University Central Hospital and University of Helsinki Biomedicum-Helsinki, Neuroscience Programme, Finland; 4 Department of Neurology, Seinäjoki Central Hospital, Finland

## Abstract

**Background:**

Fibroblast growth factor 20 (FGF20) is a neurotrophic factor preferentially expressed in the substantia nigra of rat brain and could be involved in dopaminergic neurons survival. Recently, a strong genetic association has been found between *FGF20 *gene and the risk of suffering from Parkinson's disease (PD). Our aim was to replicate this association in two independent populations.

**Methods:**

Allelic, genotypic, and haplotype frequencies of four biallelic polymorphisms were assessed in 151 sporadic PD cases and 186 controls from Greece, and 144 sporadic PD patients and 135 controls from Finland.

**Results:**

No association was found in any of the populations studied.

**Conclusion:**

Taken together, these findings suggest that common genetic variants in *FGF20 *are not a risk factor for PD in, at least, some European populations.

## Background

Parkinson's disease (PD), with a prevalence of nearly 2% in those older than 65 years of age, is one of the most common neurodegenerative disorders [1]. The cause of its clinical features (bradykinesia, rigidity, resting tremor, and postural instability) is the selective degeneration of dopaminergic neurons in the substantia nigra, leading to a deficiency of dopamine in their striatal projections areas. Although the etiology of PD is still unclear, increasing evidence suggest that genetic factors are likely to contribute [2,3]. To date, mutations in seven genes have been related to mendelian forms of the disease, and several loci across the genome have also been identified to be influencing not only familial, but also sporadic forms of PD [4-6]. One of the extant linkages to late onset PD is on chromosome 8p [7]. Analysis of candidate genes located within the region through the use of the pedigree disequilibrium test in 644 families, demonstrated a significant association between several polymorphisms at the fibroblast growth factor 20 (FGF20) and PD [8]. FGF20 is a neurotrophic factor widely expressed in dopaminergic neurons of the substantia nigra pars compacta [9], and enhances the survival of midbrain dopaminergic neurons [10].

We have attempted to replicate the previously described association by performing a case-control analysis in two different series: one from Finland and the other from Greece.

## Methods

### Patients and healthy individuals

The Finnish series is composed of 144 sporadic PD patients (mean age 67.2 years, range 38 to 88, 41% women) and 135 neurologically normal healthy control subjects (mean age 65.8 years, range 37 to 80, 64% women). Eleven patients had an onset before 45 years old. All individuals were recruited from the Neurological outpatient clinics of the Helsinki University Central Hospital and Seinäjoki Central Hospital. Patients were diagnosed according to PD Society Brain Bank criteria [11] and were either followed for at least 4 years or, alternatively, clinical follow-up for at least 2 years plus ^123^I-β-CIT-SPECT findings supporting idiopathic PD. Patients with dementia or patients who reported first-degree relatives with parkinsonism were excluded. Written informed consent was obtained from all participants. The Ethical Review Committee of the Department of Neurology at the Helsinki University Central Hospital approved the study.

The Greek series is composed of 151 sporadic PD patients (mean age 70.3, range 40 to 95, 46% women) and 186 age-, gender-and ethnicity-matched neurologically healthy controls. Three patients had an onset before 45 years old. All patients were residents of Thessaly (Central Greece) and were identified prospectively during a 3-year period (2001–2004) in the outpatient clinic for movement disorders in Larissa University Hospital and were followed up for at least one year and up to 3 years. The diagnosis of PD was based on established criteria [4].

After approval from Hospital Scientific Committee and informed consent, blood samples were drawn for DNA extraction from patients and controls.

### Genotyping

Taqman^® ^Assays-by-Design^SM ^SNP Genotyping (Applied Biosystems) based assays were employed for allelic discrimination of the two intronic SNPs (rs1989756 and rs1989754) as well as the 3'UTR SNP rs1721100. Thermal cycling and end-point PCR analysis was performed on an ABI PRISM^® ^7900HT Sequence Detection System and analyzed with SDS software (Applied Biosystems). Another SNP at the 3'UTR (rs20399075), which is located 99 base pairs (bp) from rs1721100, was genotyped using a restriction fragment length polymorphism (RFLP) approach. In brief, a PCR was conducted using the following primers: forward, 5'-AGTCTATTTTCTACTGAGAG-3'; and reverse, 5'-TAAGGACTAATCCAATGTATC-3'. The 139 bp amplified product was digested with 10 units of *Bsp*HI restriction enzyme and fragments were resolved on a 2% agarose gel stained with ethidium bromide. Two fragments of 80 bp and 59 bp appeared when a PCR fragment containing a cytosine in the polymorphic site was present.

### Statistical analysis

Odds ratios and Chi square tests were calculated using SPSS v11.0 software for windows. Linkage disequilibrium was assessed by means of a genotype association test, implemented in the Arlqeuin ver.2.000 program, and haplotype frequencies were estimated with an expectation-maximization (EM) algorithm, also implemented in the Arlequin ver.2.000 program [12]. Comparison of *FGF20 *haplotype frequencies between cases and controls was performed using Fisher's exact test. Power calculations were performed with the epidemiological program EpiInfo version 6. Pomelo Tool software  was used for multiple testing corrections.

## Results

Distribution of genotype frequencies are presented in Table 1. All polymorphisms were in Hardy-Weinberg equilibrium for PD patients and controls in both the Greek and the Finnish series. No statistically significant differences in genotype frequency were found between cases and controls in the Greek series. Allele frequencies also showed no significant association with disease in this series (data not shown). When we focused on the Finnish individuals, a slightly but not significant overrepresentation of the GG genotype in the two intronic polymorphisms (rs1989756 and rs1989754) was identified in patients compared to controls (*P *= 0.087 and 0.089 respectively). There was significant increase in the rs1989754 G allele in patients compared to control Finnish individuals (52% *vs*. 42%, *P *= 0.030). Comparison of the rs1989756, rs1721100, and ss20399075 allele frequencies did not reveal any significant differences (*P *= 0.099, *P *= 0.239, and *P *= 0.424 respectively). Repeating the analyses after the exclusion of all early onset cases did not result in any significant changes (data not shown).

**Table 1:** *FGF20 *genotype frequencies in cases and controls

	Finnish series		Greek series	
		
rs1989756	PD cases (%)	Controls (%)	PD cases (%)	Controls (%)
GG	130 (90.3)	110 (83.3)	124 (82.1)	153 (83.6)
GA	14 (9.7)	22 (16.7)	25 (16.6)	29 (15.8)
AA	0	0	2 (1.3)	1 (0.5)
	χ^2 ^(2 df); 2.928	*P *= 0.087	χ^2 ^(2 df); 0.605	*P *= 0.739
rs1989754				
GG	39 (27.1)	27 (20.1)	30 (20.0)	34 (19.2)
GC	71 (49.3)	60 (44.8)	63 (42.0)	85 (48.0)
CC	34 (23.6)	47 (35.1)	57 (38.0)	58 (32.8)
	χ^2 ^(2 df); 4.838	*P *= 0.089	χ^2 ^(2 df); 1.309	*P *= 0.520
rs1721100				
CC	74 (51.4)	68 (51.9)	73 (50.3)	90 (50.3)
CG	60 (41.7)	48 (36.6)	57 (39.3)	75 (41.9)
GG	10 (6.9)	15 (11.5)	15 (10.3)	14 (7.8)
	χ^2 ^(2 df); 1.977	*P *= 0.372	χ^2 ^(2 df); 0.702	*P *= 0.704
ss20399075				
TT	8 (6.6)	4 (3.3)	2 (1.4)	0
TC	36 (29.5)	36 (29.5)	32 (21.8)	39 (21.7)
CC	78 (63.9)	82 (67.2)	113 (76.9)	141 (78.3)
	χ^2 ^(2 df); 1.433	*P *= 0.488	χ^2 ^(2 df); 2.472	*P *= 0.291

Linkage disequilibrium (LD) analysis revealed a strong LD between all the neighboring markers (*P *values < 0.01 in all comparisons). Only the neighboring SNPs rs1721100 and ss20399075 did not show significant LD in the Finnish control group.

No significant difference in the distribution of four marker haplotypes between cases and controls was found in either of the series (see Table 2). Just a slightly decrease of the GGCC haplotype in Greek PD patients was found compared to control individuals (*P *= 0.073).

**Table 2:** *FGF20 *haplotype frequencies distributions

	Finnish sample ^a^		Greek sample ^b^	
		
	PD cases	Controls	PD cases	Controls
G G C C	42.6%	33.8%	33.8%	40.4%
G C G C	17.9%	23.4%	17.0%	19.4%
G C C C	16.1%	15.7%	21.4%	19.4%
G G C T	8.6%	8.2%	2.6%	1.9%
G C G T	8.2%	5.6%	9.2%	8.0%
A C C C	2.4%	8.3%	9.6%	8.2%
*	4.8%	5.0%	6.4%	2.7%

^a ^Fisher's exact test, *P *= 0.113.

^b ^Fisher's exact test, *P *= 0.073

* Other haplotypes with frequencies lower than 5%.

The order of SNPs are: rs1989756, rs1989754, rs1721100, and ss20399075.

## Discussion

Among all the comparisons that we have performed; only polymorphism rs1989754 G allele frequency was significantly higher in patients than controls in the Finnish sample. However, when we adjusted for multiple testing in order to control for a type I error by means of a permutation test, the *P *value was not longer significant.

Taking into account the allele frequencies, our study has a power of detecting risks from 1.7 to 3.6 in the Finnish sample and from 1.6 to 2.1 in the Greek series with 80% power (95% confidence interval). These results, thus, suggest that *FGF20 *is not a major risk factor for sporadic PD in the Finnish and Greek population.

The present work does not replicate the recently reported association between polymorphisms at the *FGF20 *gene and PD [8]. It is important to note that we have genotyped the same polymorphisms that they previously performed except the one located upstream the exon 1 (ss20399076) because this was not found in the SNP database and we could not localize this SNP based on the published report. Nevertheless, because no association was reported between this SNP and PD, the absence of this polymorphism in our work should not be a major limitation.

Several reasons could explain the discrepancy between our results and the one by Van der Walt and collaborators [8]: first, the reported positive association could be the result of a type I error, due to possible admixture. Population stratification due to ethnic admixture could have been the result of using US based sample, which was not fully detailed by the authors. Another possible explanation for the lack of replication could be the result of using different epidemiological approaches. We believe that the association reported by Walt *et al*. should be strong enough to be consistent when other genetic epidemiology methods are employed. Since the reported *p *values are in the order of 1*10E-3, one could expect a similar pattern of association, independently of the method utilized. Therefore, it seems very improbable that the lack of replication is due to the use of another methodology. A third interpretation could be that the association is real but the effect is smaller in Greek and Finnish individuals. If this was true, our series may not be large enough to assess variants that contribute to complex traits and are likely to have modest effects. Fourth and finally, a real association could exist only in groups of a specific ethnic origin and, presumably, a similar genetic background.

## Conclusion

In order to test whether the previously described association between *FGF20 *and PD is consistent in other populations, we have performed a case-control study in which we compare and analyze four biallelic polymorphisms within *FGF20 *gene in two different populations from Europe: one from Greece and one from Finland. Our results clearly indicate that genetic variations in *FGF20 *do not influence the risk of PD in these two populations. As far as we know, this is the only study that has tried to replicate the previous reported association between *FGF20 *and PD.

## Competing interests

The author(s) declare that they have no competing interests.

## Authors' contributions

JC performed the genotyping, statistical analysis, and drafted the manuscript. GX, JE, VG, OH, ED, TP, AP, GMH, and PJT, contributed to collecting materials and participated in its design and coordination together with drafting the manuscript. ABS conceived the study and participated in the conceptual design and coordination of this work, together with drafting the manuscript.

## Pre-publication history

The pre-publication history for this paper can be accessed here:


